# Prognostic impact of ICG-PDR in patients with hypoxic hepatitis

**DOI:** 10.1186/s13613-015-0092-6

**Published:** 2015-12-04

**Authors:** Thomas Horvatits, Nikolaus Kneidinger, Andreas Drolz, Kevin Roedl, Karoline Rutter, Stefan Kluge, Michael Trauner, Valentin Fuhrmann

**Affiliations:** Division of Gastroenterology and Hepatology, Department of Internal Medicine 3, Medical University of Vienna, Vienna, Austria; Department of Intensive Care Medicine, University Medical Center Hamburg-Eppendorf, Hamburg, Germany; Department of Internal Medicine V, Comprehensive Pneumology Center (CPC-M), Member of the German Center for Lung Research (DZL), University of Munich, Munich, Germany

**Keywords:** Hypoxic hepatitis, Ischemic hepatitis, Indocyanine green plasma disappearance rate, ICG-PDR

## Abstract

**Background:**

Hepatic impairment is found in up to 20 % in critically ill patients. Hypoxic/ischemic hepatitis (HH) is a diffuse hepatic damage associated with high morbidity and mortality. Indocyanine green plasma disappearance rate (ICG-PDR) is an effective tool assessing liver function in acute and chronic hepatic diseases. Aim of this study was to evaluate the prognostic impact of ICG-PDR in comparison to established parameters for risk stratification.

**Methods:**

Patients with HH were included in this prospective observational study and compared to cirrhosis, acute liver failure (ALF) and patients without underlying liver disease. ICG-PDR, measured non-invasively by finger pulse densitometry, was assessed on admission and in patients with HH serially and results were compared between groups. Diagnostic test accuracy of ICG-PDR predicting 28-day mortality was analyzed by receiver operating characteristics (ROC).

**Results:**

ICG-PDR on admission was significantly lower in patients with liver diseases than in patients without hepatic impairment (median 5.7 %/min, IQR 3.8–7.9 vs. 20.7 %/min, IQR 14.1–25.4 %/min; *p* < 0.001). ICG-PDR predicted 28-day mortality independently of SOFA score and serum lactate in patients with underlying liver disease (HR 1.27, 95 % CI 1.10–1.45, *p* < 0.001). In patients with HH, ICG-PDR was identified as best predictor of 28-day mortality which performed significantly better than SOFA, lactate, INR and AST over course of time (*p* < 0.05). Best cut-off for identification of 28-day survivors was ICG-PDR ≥9.0 %/min 48 h after admission.

**Conclusions:**

ICG-PDR is an independent predictor of mortality in patients with liver disease. Diagnostic test accuracy of ICG-PDR was superior to standard liver function parameters and established scoring systems in patients with HH.

## Background

Hepatic impairment is a frequent finding in critically ill patients. Cholestatic liver dysfunction is found in up to 20 %, whereas hypoxic liver injury is found in up to 10 % of patients admitted to medical intensive care units (ICU) [1, 2]. Both, cholestatic as well as hypoxic liver dysfunction is associated with increased mortality [3–5]. Acute liver failure (ALF) is a life-threatening but rare disease, with an incidence of less than 10 cases/million persons [6]. Acute-on chronic liver failure, defined as acute decompensation of cirrhosis, is characterized by rapid progression and high mortality rates [7].

Hypoxic hepatitis (HH), also known as ischemic hepatitis or shock liver, is a diffuse hepatic damage characterized by centrilobular liver cell necrosis associated with high morbidity and mortality [2, 4, 5]. HH is defined as a sharp increase of aminotransferase levels in an acute setting of cardiac, septic or respiratory failure. HH is associated with high mortality rates up to more than 50 % [2, 5, 8–10]. Laboratory parameters as serum aspartate aminotransferase level (AST), international normalized ratio (INR) and arterial serum lactate have been described to be elevated in HH non-survivors [9]. However, there is still a lack of prognostic tool for risk stratification in patients with HH.

The fluorescent medical dye indocyanine green (ICG) is taken up exclusively by hepatocytes and is secreted unchanged into the bile. As ICG is removed solely by the liver without entering enterohepatic circulation, it has been described as an effective quantitative liver function test [11, 12]. Elimination of ICG represents both, hepatic blood flow as well as parenchymal function. ICG clearance has been described for assessing liver function in patients undergoing liver surgery (hepatic resection in patients with hepatocellular carcinoma (HCC)) as well as in donors and recipients of living donor liver transplantation (OLT) [13–15]. Furthermore, indocyanine green plasma disappearance rate (ICG-PDR) has been evaluated as prognostic marker in patients with advanced liver cirrhosis [16, 17]. Recently, ICG clearance has been described as an effective tool for assessing portal hypertension in patients with compensated liver cirrhosis [18]. Additionally, ICG-PDR serves as a good predictor for mortality in critically ill patients [12, 19–21].

However, there is a lack of data regarding ICG clearance in critically ill patients with hepatic impairment. In particular, there are no data regarding ICG clearance in patients with HH available.

This study evaluates the prognostic impact of ICG-PDR, in comparison to established parameters for risk stratification, in patients with HH and compares it to patients with ALF, decompensated liver cirrhosis or without underlying liver disease at the ICU.

## Patients and methods

### Patients

In this observational study, impact of ICG-PDR was prospectively evaluated and compared to established parameters for risk stratification. Patients with HH were compared to patients with ALF or liver cirrhosis admitted to the ICU and critically ill patients suffering from severe shock but without liver disease. Exclusion criteria were age <18, pregnancy, HCC, previous OLT, presence of extracorporeal life support system, and presence of hyperthyroidism and iodine allergy according manufacturers product information. ICG-PDR was assessed in between therapy cycles in patients requiring renal replacement therapy (RRT).

Data collection was performed on daily basis. Sequential Organ Failure Assessment (SOFA) [22], Simplified Acute Physiology Score II (SAPSII) [23], Model of End-stage Liver Disease (MELD) [24] were calculated on ICU admission as well as 24 and 48 h thereafter. MELD score was calculated for patients with HH and controls as well. However, MELD score is routinely used mainly in patients with cirrhosis and ALF. Shock index (SI) was calculated as ratio of heart rate and systolic blood pressure as established representative of systemic hemodynamics [25]. Vasopressor support and dosage were recorded. 28-day mortality was prospectively assessed.

The study protocol was approved by the Ethics Committee of the Medical University of Vienna and performed according to the ethical principles of the revised Helsinki declaration. Patients’ informed consent was obtained.

### Definitions

Diagnosis of HH was made according the well-established criteria: (a) setting of cardiac, circulatory or respiratory failure, (b) dramatic but transient elevation in serum AST levels to at least 20-fold the upper limit of normal, (c) exclusion of other putative causes of liver cell necrosis (viral or drug induced hepatitis) [8]. Patients with HH accompanying cirrhosis were excluded from the study.

Liver cirrhosis was diagnosed by histological or by combination of clinical signs and laboratory findings or by typical radiologic signs in abdominal ultrasonography or computed tomography scan.

Acute liver failure was defined according to the international consensus as INR >1.5 and the presence of hepatic encephalopathy in patients without liver cirrhosis and duration of the disease (jaundice) of less than 26 weeks [26].

### Measurement of ICG-PDR

For each measurement ICG (0.25 mg/kg bodyweight) was injected intravenously via a central line followed by 5 ml of normal saline, as described elsewhere [27]. Plasma disappearance rate of ICG was measured non-invasively by using a finger pulse densitometry system (LiMON, Pulsion Medical Systems SE, Munich, Germany) [28] (normal range of ICG-PDR is 18–25 %/min). ICG-PDR was assessed in all patients on admission and in patients with HH serially on a daily basis until day 5. ICG measurement was not possible due to poor peripheral perfusion as consequence of severe shock in two patients with HH.

### Management

Patients with septic or cardiogenic shock were treated according to standardized protocols [29, 30]. Intravenous fluid administration as well as vasopressor therapy was initiated in patients meeting shock criteria aiming to maintain a mean arterial blood pressure of >65 mmHg. Early initiation of broadspectrum antibiotic treatment was performed according standardized protocols [29]. Antimicrobial therapy was adapted to culture results. RRT was performed in patients with renal failure and/or metabolic acidosis. Patients with suspected acetaminophen-associated ALF received intravenous *N*-acetylcysteine [31].

### Data analysis and statistics

Data were described as median and 25–75 % interquartile range (IQR). Metric variables were compared using Mann–Whitney U test and dichotomous variables were compared using Chi-square analysis. Correlation analysis was performed using Spearman’s correlation. Cox regression proportional hazard analysis was performed to assess predictors of 28-day mortality. A forward stepwise procedure was used to identify most potent predictors.

The overall diagnostic test accuracy of ICG-PDR, SOFA, arterial serum lactate, AST levels and INR was assessed by receiver operating characteristics (ROC) expressed as their area under the curve (AUROC). We compared AUROCs at several time points using standard non-parametric methods. Estimates of diagnostic test accuracy (sensitivity, specificity) were calculated using standard methods. One-way repeated/multiple measured analysis of variance (ANOVA) was performed in patients with HH surviving until day 5. A Greenhouse-Geisser correction was used for sphericity. For data management and analyses, we used MS Excel 2008 for Mac, SPSS 21 for Mac (SPSS, Inc. Chicago, IL, USA), and Stata 12 for Mac (Stata Corp., College Station, TX, USA). All *p* values reported are two sided and *p* < 0.05 was considered significant.

## Results

### Patients’ characteristics

A total of 97 patients were included in this observational study.

This cohort comprised 52 patients with HH, 35 patients with cirrhosis admitted to the ICU because of acute decompensation and 10 patients with ALF.

Main reasons for occurrence of HH were cardiogenic (*n* = 32) and septic *n* = 20) shock. Only the first episode of HH that occurred during the stay at the ICU was assessed in this study.

Cause of ALF was acetaminophen intoxication (*n* = 3), non-acetaminophen drug induced (*n* = 2), or occurred as consequence of viral hepatitis (*n* = 2) and unknown reasons (*n* = 3).

The most common cause of cirrhosis was alcoholic liver disease (*n* = 18), followed by viral hepatitis (*n* = 12) and others (*n* = 5). The main ICU-admission diagnosis in patients with cirrhosis was sepsis/septic shock (*n* = 14) followed by coma hepaticum/hepatic encephalopathy grade 3–4 (*n* = 11) and hemorrhagic shock due to gastrointestinal bleeding (*n* = 10).

The control group included 22 critically ill patients. Main admission diagnoses were cardiogenic (*n* = 12) and septic (*n* = 10) shock without signs of severe acute liver injury, viral or drug induced hepatitis or cirrhosis. Detailed clinical characteristics of the patients are illustrated in Table 1.

**Table 1:** Patient characteristics

	Patients with liver disease	HH	ALF	Cirrhosis	Control
Parameter					
*n*	97	52	10	35	22
Male, (%)	61 (63)	34 (65)	4 (40)	23 (66)	16 (73)
Age, years^a, b, e^	54 (44–66)	64 (47–76)	40 (35–49)	48 (39–57)	53 (44–67)
SOFA^b, c^	12 (8–16)	13 (9–16.8)	9 (5.8–14)	11 (7–14)	10.5 (7–13)
SAPS II^a, b, c^	57 (38–71)	60.5 (43.3–78.5)	26.5 (15.8–56.5)	51 (35–67)	50 (36–59)
MELD^a, c, d, e, f^	22 (17–30)	21.5 (15–28.8)	31 (24.5–35.3)	19 (17–30)	8 (6–14.5)
Mechanical ventilation, (%)^e, f^	75 (77)	42 (81)	7 (70)	26 (74)	21 (96)
Vasopressor, (%)^a^	74 (76)	45 (87)	4 (50)	24 (69)	17 (77)
Mean arterial pressure, mmHg	72 (65.5–83.5)	71.5 (68.3–77)	75.5 (66.8–88.3)	70 (65–80)	72 (65.5–83.5)
Heart rate, beat/min	94 (80–111.5)	95 (83–113.8)	93.5 (83.3–105)	92 (80–113)	90 (76.8–105)
Renal replacement therapy, (%)	39 (40)	21 (40)	6 (60)	12 (34)	5 (23)
28-day mortality, (%)^a, d^	48 (49)	27 (52)	1 (10)	20 (57)	7 (32)

Data are shown as median and IQR or as number and percentage

^a^ *p* < 0.05 HH vs. ALF

^b^ *p* < 0.05 HH vs. cirrhosis

^c^ *p* < 0.05 HH vs. control

^d^ *p* < 0.05 ALF vs. cirrhosis

^e^ *p* < 0.05 ALF vs. control

^f^ *p* < 0.05 cirrhosis vs. control

SOFA score as well as SAPS II score on admission were significantly higher in patients with HH compared to cirrhosis and controls (*p* < 0.05). MELD score was significantly elevated in patients with HH, cirrhosis and ALF in comparison to control patients (*p* < 0.001). Vasopressor support and hemodynamics were comparable between controls and patients with HH as shown in Table 1. ICG-PDR did not correlate with SI, norepinephrine or dobutamine dose neither in the overall group of patients with underlying liver disease (spearman’s *r* = 0.03, *p* = n.s.; *r* = 0.01, *p* = n.s.; *r* = 0.17, *p* = n.s.) nor in patients with HH (spearman’s *r* = 0.09, *p* = n.s.; *r* = −0.08, *p* = n.s.; *r* = 0.15, *p* = n.s.), respectively. Furthermore, ICG-PDR did neither correlate with CVP (central venous pressure) (spearman’s *r* = −0.1, *p* = n.s.), central venous oxygen saturation, (spearman’s *r* = 0.02, *p* = n.s.) nor with PaO_2_/FiO_2_ ratio (spearman’s *r* = −0.1, *p* = n.s.).

Aminotransferase levels on admission were significantly higher in patients with HH in comparison to patients with liver cirrhosis or patients without liver disease (*p* < 0.001). Furthermore, highest arterial serum lactate levels were found in patients with HH and ALF. Patients with HH showed significantly lower serum bilirubin levels than patients with cirrhosis and ALF (*p* < 0.001). ICG-PDR correlated with bilirubin in the overall cohort of patients with underlying liver disease as well as in patients with HH (spearman’s *r* = −0.47, *p* < 0.001; *r* = −0.48, *p* < 0.001). Detailed laboratory data are illustrated in Table 2. Median ICU length of stay was 9 days (IQR 4–15 days), whereas median hospital length of stay was 13 days (IQR 6–27 days). One patient with ALF underwent OLT.

**Table 2:** Laboratory characteristics

	Patients with liver disease	HH	ALF	Cirrhosis	Control
Parameter					
*n*	97	52	10	35	22
ICG-PDR, %/min^b, c, e, f^	5.7 (3.8–7.9)	7 (4.7–8.9)	5.7 (4.8–8.0)	4.1 (3.4–5.9)	20.7 (14.1–25.4)
Bilirubin, mg/dl^a, b, c, e, f^	3.7 (1.7–9.7)	2.2 (0.8–3.8)	23.8 (9.1–29.2)	7.7 (3.3–16.6)	0.8 (0.6–1.3)
INR^a, c, d, e, f^	1.8 (1.5–2.3)	1.7 (1.4–2.3)	3.3 (2.1–5.2)	1.7 (1.4–2.2)	1.2 (1.1–1.3)
AST, U/l^b, c, d, e^	1096 (133–3798)	2667 (1267–5015)	1815 (216–5289)	84 (55–202)	67 (38–418)
ALT, U/l^b, c, d, e^	743 (76–2119)	1430 (871–2417)	2224 (202–6173)	43 (24–93)	54 (21–176)
Lactate, mmol/l^b^	3.5 (2–6.2)	4 (2.3–9.3)	4 (2.3–5.3)	2.5 (1.8–4)	3 (1.8–3.6)
Creatinine, mg/dl^a, c^	1.5 (1–2.4)	1.7 (1.3–2.3)	0.9 (0.7–1.5)	1.2 (0.8–3.1)	1.1 (0.8–2)

Data are shown as median and IQR

*AST* aspartate aminotransferase level, *ALT* alanine aminotransferase level, *INR *international normalized ratio

^a^ *p* < 0.05 HH vs. ALF

^b^ *p* < 0.05 HH vs. cirrhosis

^c^ *p* < 0.05 HH vs. control

^d^ *p* < 0.05 ALF vs. cirrhosis

^e^ *p* < 0.05 ALF vs. control

^f^ *p* < 0.05 cirrhosis vs. control

### ICG-PDR on admission and outcome in critically patients with liver diseases

**Fig. 1:**
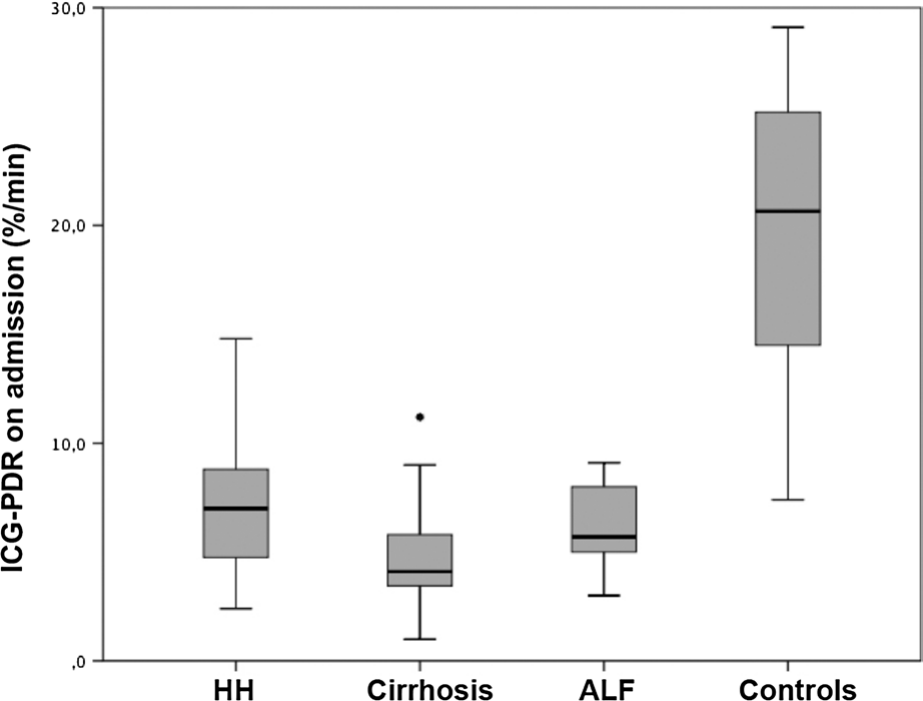
ICG-PDR on admission in different liver disease entities. HH, liver cirrhosis, ALF vs. patients without hepatic impairment (median 5.7 %/min, IQR 3.8–7.9 vs. 20.7 %/min, IQR 14.1–25.4 %/min; *p* < 0.001). *Box plot* marks median, interquartile ranges and extreme values. *HH* hypoxic hepatitis, *ALF* acute liver failure

ICG-PDR on admission was significantly lower in patients with liver diseases (HH, liver cirrhosis, ALF) than in the control group without hepatic impairment (median 5.7 %/min, IQR 3.8–7.9 vs. 20.7 %/min, IQR 14.1–25.4 %/min; *p* < 0.001) as illustrated in Fig. 1.

There was no significant difference of ICG-PDR between HH and ALF. Detailed data of ICG-PDR is shown in Table 2.

ICG-PDR was significantly higher in 28-day survivors compared to non-survivors in the overall group of patients with liver disease (median 6.6 %/min, IQR 4.8–9.1 %/min vs. median 4.9 %/min, IQR 3.4–6.6 %/min; *p* < 0.001) as well as in the group of patients with HH (median 8.6 %/min, IQR 6.3–10.5 %/min vs. median 5.4, IQR 3.5–7.2 %/min, *p* < 0.001).

Univariate cox regression analysis detected a significant association of ICG-PDR, SOFA score and arterial serum lactate on admission with 28-day mortality (HR 1.31, 95 % CI 1.15–1.50, *p* < 0.001; HR 1.12, 95 % CI 1.04–1.19, *p* < 0.001; HR 1.12, 95 % CI 1.07–1.18, *p* < 0.001) in patients with liver disease. In contrast, there was no association of age, sex, aminotransferase levels, INR and bilirubin levels with 28-day mortality. ICG-PDR remained an independent predictor of 28-day mortality even after correction with SOFA score and arterial serum lactate in multivariate cox regression model (HR 1.27, 95 % CI 1.10–1.45, *p* < 0.001). ROC analysis revealed that ICG-PDR had the highest prediction of 28-day mortality, followed by arterial serum lactate and SOFA (Table 3).

**Table 3:** ROC analysis of various parameters assessed on ICU admission for prediction of 28-day mortality in patients with underlying liver disease

Parameter	AUROC	95 % CI
ICG-PDR^a^	0.73	0.63–0.83
Lactate^a^	0.72	0.62–0.83
SOFA^b^	0.69	0.58–0.79
MELD	0.65	0.54–0.76
INR	0.62	0.51–0.73
Bilirubin	0.54	0.43–0.66
AST	0.54	0.42–0.66

*AST* aspartate aminotransferase level, *INR* international normalized ratio

^a^AUROC *p* < 0.05 ICG-PDR, Lactate vs. Bilirubin, AST

^b^AUROC *p* < 0.05 SOFA vs. AST

### ICG-PDR and outcome in patients with hypoxic hepatitis

**Fig. 2:**
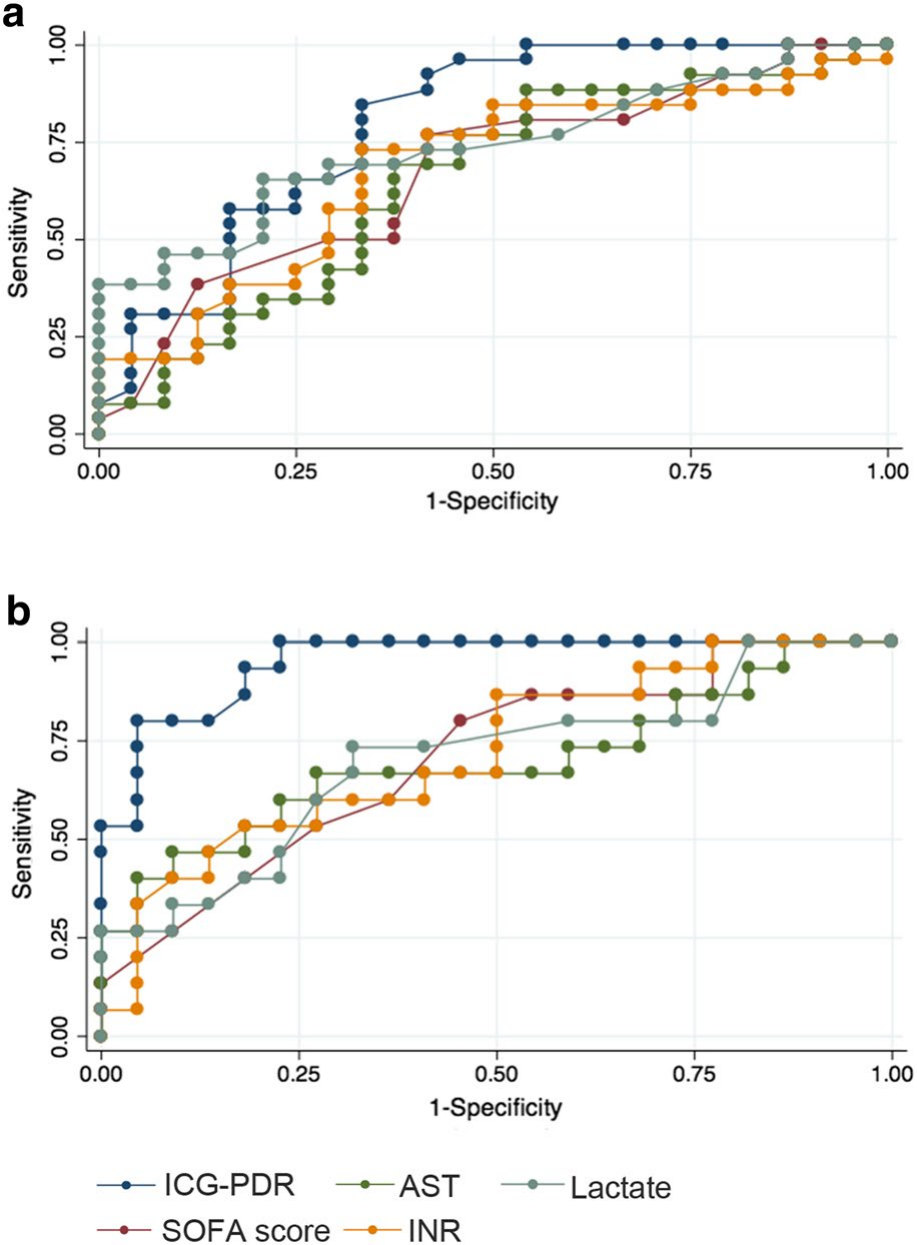
ROC predicting 28-day mortality in patients with HH **a** on admission and **b** 48 h after admission. *AST* aspartate aminotransferase level, *INR* international normalized ratio

ICG-PDR had highest AUROC of all parameters assessed on admission in regard to 28-day mortality as shown in Fig. 2. 48 h after admission, AUROC of ICG-PDR was significantly higher than AUROC of SOFA, arterial serum lactate, INR and AST (*p* < 0.05; see Table 4). AUROC of Δ-ICG as increase of ICG-PDR from admission to 48 h value was 0.85.

**Table 4:** ROC analysis for predicting 28-day mortality in patients with HH

Parameter	AUROC	95 % CI
BL-ICG-PDR	0.81	0.68–0.93
24-ICG-PDR^a^	0.88	0.76–0.99
48-ICG-PDR^b^	0.96	0.90–1.00
BL-Lactate	0.74	0.60–0.88
24-Lactate	0.70	0.53–0.87
48-Lactate^b^	0.71	0.54–0.88
BL-SOFA	0.69	0.54–0.83
24-SOFA	0.68	0.52–0.83
48-SOFA^b^	0.73	0.58–0.89
BL-INR	0.66	0.51–0.81
24-INR^a^	0.69	0.53–0.85
48-INR^b^	0.76	0.61–0.91
BL-AST	0.63	0.48–0.78
24-AST	0.76	0.61–0.90
48-AST^b^	0.72	0.54–0.89

*AST* aspartate aminotransferase level, *INR* international normalized ratio

^a^AUROC *p* < 0.05 24-ICG-PDR vs. 24-INR

^b^AUROC *p* < 0.05 48-ICG-PDR vs. 48-Lactate, 48-SOFA, 48-INR, 48-AST

Youden index showed best discrimination of 28-day survivors and non-survivors at a cut-off of ICG-PDR ≥7.8 %/min on admission (sensitivity of 68 %, specificity of 85 %) and ≥9.0 %/min 48 h after admission (sensitivity 79 %, specificity 100 %).

**Fig. 3:**
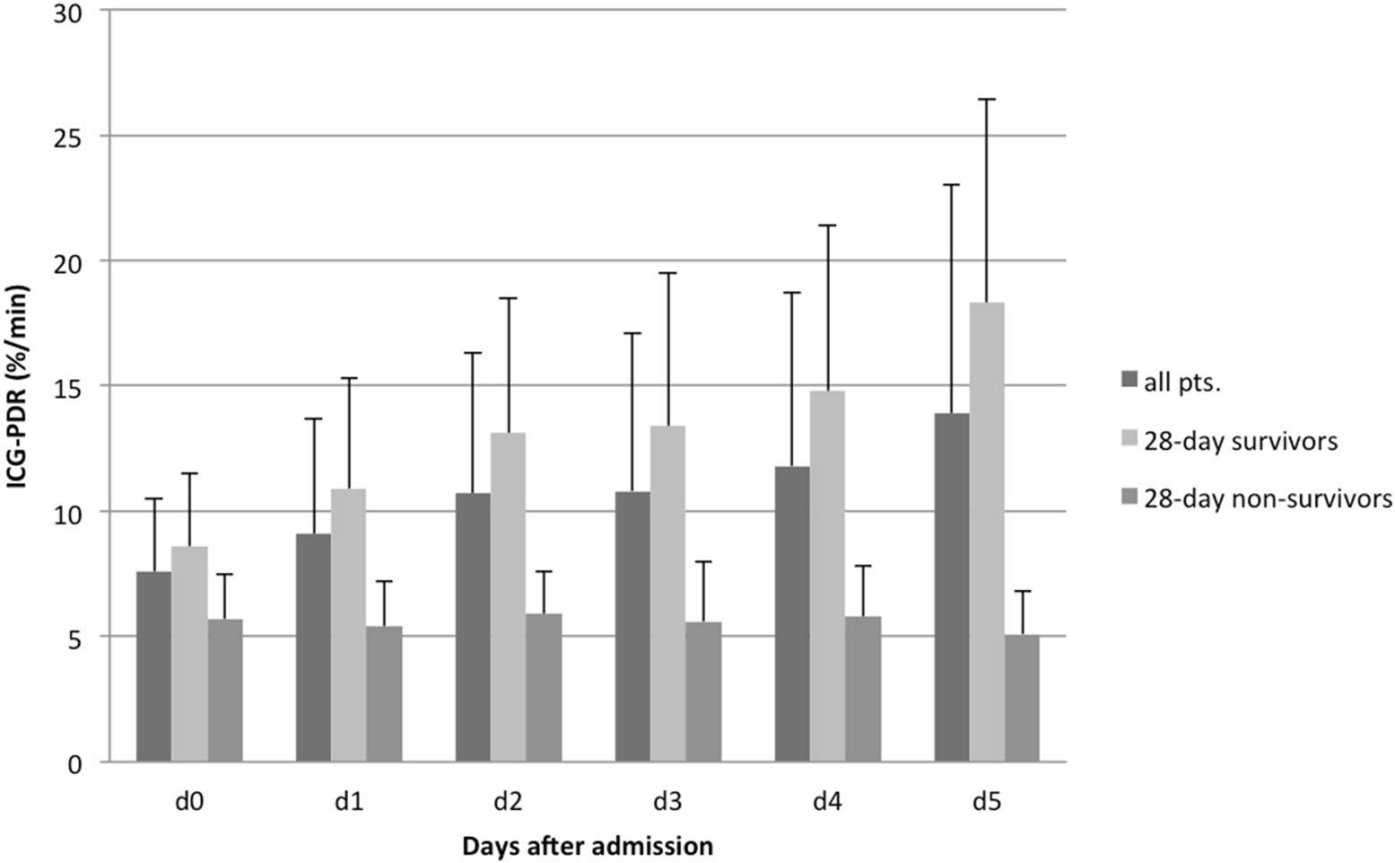
Time course of ICG-PDR in patients with HH. ICG-PDR measurement on 4 consecutive days after admission in 30 patients with HH that survived until day 5. Repeated measures ANOVA showed a significant effect of time of ICG-PDR in 28-day survivors (*p* < 0.001)

Change of ICG-PDR over time in 30 patients with HH is illustrated in Fig. 3. One way repeated measured analysis of variance (ANOVA) showed a significant effect of ICG-PDR over time in 28-day survivors (Greenhouse-Geisser corrected *F* = 22.4, *df* = 2.5, *p* < 0.001) but not in 28-day non-survivors (*p* = n.s.).

## Discussion

Hepatic impairment by means of cholestatic or hypoxic liver disease is of pivotal prognostic relevance in critically ill patients. In particular, HH seems to contribute to dramatically high mortality rates [2, 4, 5, 8, 9]. Beside laboratory parameters, different quantitative liver function tests such as ICG clearance or maximal liver function capacity test (LiMAx) or tests for evaluation of hepatic blood flow (galactose clearance) aim to facilitate assessment of liver function in acute and chronic hepatic impairment [4, 32, 33]. ICG-PDR is reported as prognostic parameter in stable patients with cirrhosis and in critically ill patients without underlying liver disease [17–21]. The LiMAx test was described as prognostic marker in liver transplant candidates as well as in patients with sepsis-related liver failure [32, 33]. However, none of the quantitative liver function tests were evaluated in the setting of acute hypoxic liver injury. Therefore, this study evaluates the prognostic impact of ICG-PDR in critically ill patients with HH compared to other forms of acute and chronic liver failure.

We could demonstrate that the degree of reduced ICG-PDR was comparable in patients with HH and patients with ALF. In contrast, control patients with similar severity of shock without evident hepatic impairment had significantly higher ICG-PDR. ICG-PDR levels in critically ill patients with cirrhosis were consistent with previous studies [17].

Furthermore, we could demonstrate a strong association of ICG-PDR with 28-day mortality in critically ill patients with liver disease. Best prediction of 28-day mortality was found in patients with HH. Additionally, diagnostic test accuracy of ICG-PDR was increasing over time in patients with HH. 48 h after admission ICG clearance performed significantly better than SOFA score, arterial serum lactate, INR or AST in prediction of 28-day mortality. A previous study identified INR as well as SOFA score as independent predictors for overall mortality in patients with HH [9]. Diagnostic test accuracy of ICG-PDR was superior compared to standard liver function parameters as well as SOFA score in our cohort. Furthermore, the dynamics of ICG-PDR over time demonstrated that ICG clearance did not increase over the course of time in patients that died within the first 28 days, whereas a constant increase was observed in survivors (see Fig. 3). Therefore, assessment of ICG-PDR during the course of the disease seems to be of particular prognostic relevance. 28-day survivors could be identified using a cut-off ICG-PDR ≥9.0 %/min 48 h after admission with sensitivity of 77 % and specificity 100 %.

ICG clearance is frequently used as global marker of hepatic function as it illustrates hepatic purification and also hepatic blood flow [11]. ICG-PDR did not correlate with SI, CVP, norepinephrine or dobutamine dose, respectively. Furthermore, the control group of our cohort with comparable etiology and severity of shock but without HH or any other cause of acute or chronic liver dysfunction had significantly higher ICG-PDR levels, indicating a strong association of ICG-PDR and hepatic function also in patients with severe shock. This is in accordance with another study that observed no association of alterations of cardiac output and a change in flow-dependent hepatic function with respect to ICG-PDR in critically ill patients without clinical manifestation of hepatic impairment [34].

There are some limitations of our study. First, the sample size of our study was rather small. However, this is the first prospective study investigating the prognostic impact of ICG-PDR in critically ill patients with life-threatening acute and chronic liver diseases. Second, this study was conducted at a medical ICU of a university hospital. Thus, conclusions drawn in this study may not be generalizable to patients treated in surgical or tertiary care hospital wards.

## Conclusions

In summary, we could demonstrate that ICG-PDR is a strong and independent predictor of 28-day mortality in critically ill patients with underlying liver disease. Diagnostic test accuracy of ICG-PDR was superior to standard liver function parameters and well-established scoring systems in patients with HH, which increased over course of time. ICG-PDR seems to be a bedside feasible non-invasive tool for early risk stratification in patients with HH.

## Abbreviations

HH: hypoxic hepatitis
ICU: intensive care unit
AST: aspartate aminotransferase level
INR: international normalized ratio
ICG: indocyanine green
ICG-PDR: indocyanine green plasma disappearance rate
ALF: acute liver failure
HCC: hepatocellular carcinoma
RRT: renal replacement therapy
OLT: liver transplantation
SOFA: Sequential Organ Failure Assessment
SAPSII: Simplified Acute Physiology Score II
MELD: model of end-stage liver disease
SI: shock index
HE: hepatic encephalopathy
IQR: interquartile range
ROC: receiver operating characteristics
AUROC: area under the ROC curve
ANOVA: analysis of variance
CVP: central venous pressure
